# Investigation on In Situ Carbon-Coated ZnFe_2_O_4_ as Advanced Anode Material for Li-Ion Batteries

**DOI:** 10.3390/gels8050305

**Published:** 2022-05-16

**Authors:** Mir Waqas Alam, Amal BaQais, Mohammed M. Rahman, Muhammad Aamir, Alaaedeen Abuzir, Shehla Mushtaq, Muhammad Nasir Amin, Muhammad Shuaib Khan

**Affiliations:** 1 Department of Physics, College of Science, King Faisal University, Al Ahsa 31982, Saudi Arabia; aabuzir@kfu.edu.sa; 2 Department of Chemistry, College of Science, Princess Nourah Bint Abdulrahman University, Riyadh 11671, Saudi Arabia; 3 Department of Chemistry & CEAMR, King Abdulaziz University, Jeddah 21589, Saudi Arabia; mmrahman@kau.edu.sa; 4 Department of Basic Science, Preparatory Year Deanship, King Faisal University, Al Ahsa 31982, Saudi Arabia; msadiq@kfu.edu.sa; 5 School of Natural Sciences, National University of Sciences & Technology, Islamabad 44000, Pakistan; shehla.mushtaq@sns.nust.edu.pk; 6 Department of Civil and Environmental Engineering, College of Engineering, King Faisal University, Al Ahsa 31982, Saudi Arabia; mgadir@kfu.edu.sa; 7 International Research Center for Renewable Energy (IRCRE), State Key Laboratory of Multiphase Flow in Power Engineering (MPFE), Xi’an Jiaotong University, 28 West Xianning Road, Xi’an 710049, China; m.shuaibkhan@mail.xjtu.edu.cn

**Keywords:** zinc ferrate, carbon-coated, sol–gel, anode material, Li-ion storage

## Abstract

ZnFe_2_O_4_ as an anode that is believed to attractive. Due to its large theoretical capacity, this electrode is ideal for Lithium-ion batteries. However, the performance of ZnFe_2_O_4_ while charging and discharging is limited by its volume growth. In the present study, carbon-coated ZnFe_2_O_4_ is synthesized by the sol–gel method. Carbon is coated on the spherical surface of ZnFe_2_O_4_ by in situ coating. In situ carbon coating alleviates volume expansion during electrochemical performance and Lithium-ion mobility is accelerated, and electron transit is accelerated; thus, carbon-coated ZnFe_2_O_4_ show good electrochemical performance. After 50 cycles at a current density of 0.1 A·g^−1^, the battery had a discharge capacity of 1312 mAh·g^−1^ and a capacity of roughly 1220 mAh·g^−1^. The performance of carbon-coated ZnFe_2_O_4_ as an improved anode is electrochemically used for Li-ion energy storage applications.

## 1. Introduction

Based on their high specific capacity, lightweight feature and small volumes, Li-ion batteries are observed as promising energy storage devices. Since it was commercially introduced by Sony in 1990, it received explosive development in the field of portable electronics such as smart-phones, laptops and other wearable electronic devices. The design of high-energy electrode materials should be the primary focus of researchers in order to extend its application to the field of electric car and large-scale energy grid storage. Graphite is a commercially available anode material for Li-ion batteries due to their low cost, long cycle life and environmental friendliness. In spite of these advantages, the lower theoretical capacity of 372 mAh·g^−1^ along with potential safety concerns of dendrite formation and short-circuiting forced researchers to find alternative anode materials to satisfy the growing demand of LIBs [1,2,3,4,5,6,7,8,9,10].

Transition metal oxides of the form MxOy [11,12,13,14,15] (M = Mn, Co, Ni, Fe, etc.) are studied as alternative anode materials due to their larger theoretical capacity and higher Li intercalation potential than commercial graphite. The pioneering work of Poizot et al. in 2000 provided the impetus for the application of 3d TMOs as anode material. Currently, binary and ternary transition metal oxides combining different 3D transition metals are studied due to their higher theoretical capacity that is greater than commercial graphite electrodes. Moreover, a suitable combination of transition metal oxides results in better electrochemical performance and electronic conductivity [16,17,18]. Li storage capacity in this material is achieved through a reversible reaction between the Li-ion and metal oxide, which forms nanocrystals of metals scattered in the Li_2_O matrix. Continuous Li insertion and exertion cause large volume change, resulting in the pulverization of the anode. As a consequence, the electrical connectivity between the active anode materials breaks down, causing severe capacity fade over prolonged cycles. Two different techniques are adopted by the researchers to overcome this drawback. One is to synthesize nanoparticles with different morphologies such as nanoparticles, nanorods [19,20], nanosphere [21,22], nanospindles [23,24], nanowires [25,26], TiO_2_-B [27] and ZnAl_2_O_4_ [28]. These nanostructured materials could better accommodate strains caused by Li insertion and extraction by reducing the transport path of ions and electrons. This high surface area could induce Solid Electrolyte Interface (SEI) thick-layer formation, which consumes more Li ions resulting in irreversible capacity loss during initial cycles [27,28,29,30]. Shashan Yao et al. reported CoFe_2_O_4_ as an electrocatalyst for Li batteries [29,30].

Secondly, carbon coating is the most widely used technique to protect the inner active material from side reactions and maintains its high capacity. This layer acts as a buffer medium to volume changes and provides better electrical conductivity for good stability. Iron-based transition metal oxides are receiving more attention because of their natural abundance, non-toxicity low cost and environmental friendliness. Specifically, ZnFe_2_O_4_ is studied more widely studied, in which divalent and trivalent ions occupy tetrahedral A and octahedral B sites. It has a high theoretical capacity (1072 mAh·g^−1^) arising from both conversion and alloying reactions. Its lower working voltage of 1.5 V for Li insertion and extraction is useful in achieving high energy density. In addition to these advantages, it still suffers from severe capacity fade, poor electronic conductivity and large volume changes during Li insertion and extraction. Different approaches were used by researchers to mitigate these problems [31,32,33].

Here, ZnFe_2_O_4_ is synthesized using the facile sol–gel method. The sol–gel method is considered effective for modifying the surface of substrates. Obtaining a high surface area and stable surfaces is the most important advantage of the sol–gel method. As a source of both carbon and chelating agents, citric acid is used. The presence of carbon content is effectively controlled by varying the concentration of citric acid, and its impact on the electrochemical performance is studied. The optimized sample is studied by cyclic, galvanostatic and electrical impedances. The results are impressive with a high capacity at a current density of 100 mA·g^−1^.The capacity is still maintained above 1100 after 50 cycles with very good stability. The results show that carbon-coated ZnFe_2_O_4_ will be a cost effective and highly stable anode for Li ion batteries.

## 2. Results and Discussion

**Figure 1:**
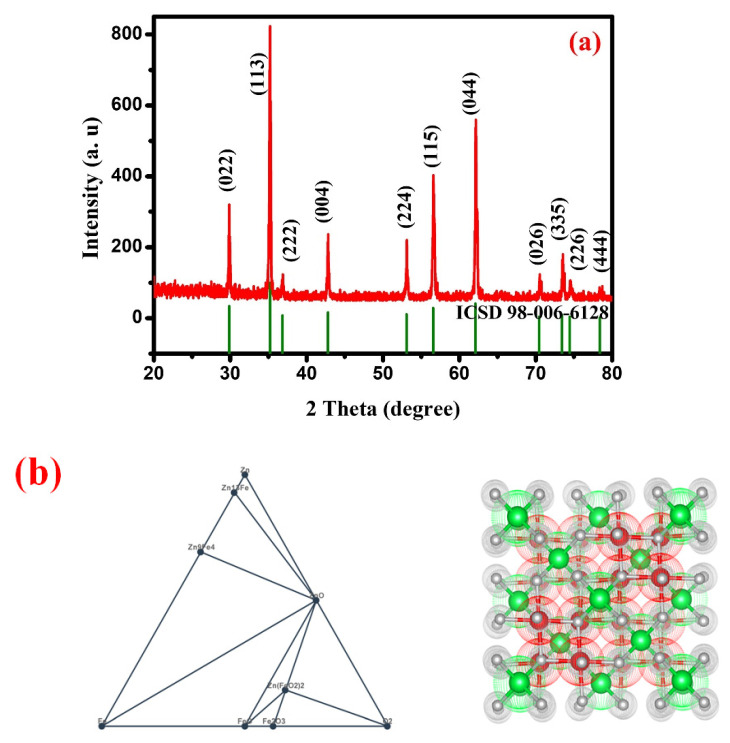
(**a**) The XRD patterns of Carbon coated ZnFe_2_O. (**b**) The simulated structure of ZnFe_2_O_4_.

The XRD patterns of in situ carbon-coated ZnFe_2_O_4_ are shown in Figure 1 As shown in Figure 1a, all peaks are well indexed and the diffraction peaks at angles of 17.98°, 29.6°, 35.14°, 36°, 42.72°, 52.78°, 56.32°, 61.96°, 70.61° and 73.57°, which corresponds to the hkl plane of (111), (220), (311), (222), (400), (422), (511), (440), (620) and (533), respectively. The XRD patterns show that the sample possesses a cubic spinel structure. All indexed peaks and intensity wells match with the standard ICSD 98-006-6128 [34,35]. The well-indexed highest point shows that the prepared sample has a good crystalline nature. The carbon peak is not observed and remains in an amorphous nature. Thus, in situ carbon coated ZnFe_2_O_4_ was successfully synthesized without affecting the basic nature of ZnFe_2_O_4_. The simulated structure of ZnFeO_4_ is shown in Figure 1b.

**Figure 2:**
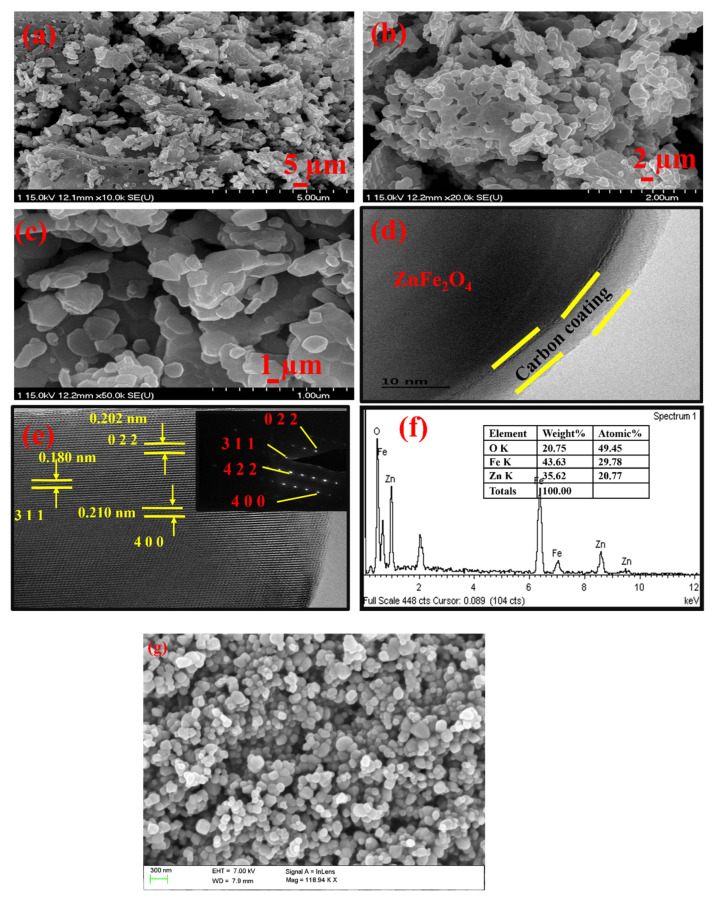
FESEM images of in situ carbon-coated ZnFe_2_O_4_ (**a**) with 5 µm, (**b**) with 2 µm and (**c**) with 1 µm. (**d**,**e**) HRTEM and SAED images of ZnFe_2_O_4_. (**f**) EDAX. (**g**) SEM images of ZnFe_2_O_4_ without carbon coatings.

Figure 2 shows FESEM and HRTEM images of in situ carbon-coated ZnFe_2_O_4_. Morphological analysis was performed to study the structural nature of the prepared samples. Figure 2a–c show the FESEM images of the in situ carbon-coated ZnFe_2_O_4_. An agglomeration with unevenly distributed particles was formed. The range of the particle size is about 100–200 nm. Upon observing Figure 2b, irregular particles have been formed with various sizes, accumulation and uneven spread due to the combustion process produced by carbon agglomeration. Carbon was coated on the sample by adding citric acid as a chelating agent for the combustion method. The carbon source itself acted as a carbon source for the prepared samples. Figure 2c shows the uneven spherical structure. The uneven nature may be due to the presence of carbon on the sample. Figure 2d shows that in situ carbon was coated on the surface of ZnFe_2_O_4_, which indicates that the carbon-coated ZnFe_2_O_4_ was successfully synthesized. The conductive carbon on the outer surface greatly increases the performance of the electrode during electrochemical analysis. The SAED model for the prepared samples is shown in Figure 2e. The clear points show that the sample was purely crystalline, without any contamination particles. Figure 2f demonstrates the EDAX spectra of the prepared samples to confirm the presence of the elements. The thickness of the carbon coating is about 2–3 nm.

**Figure 3:**
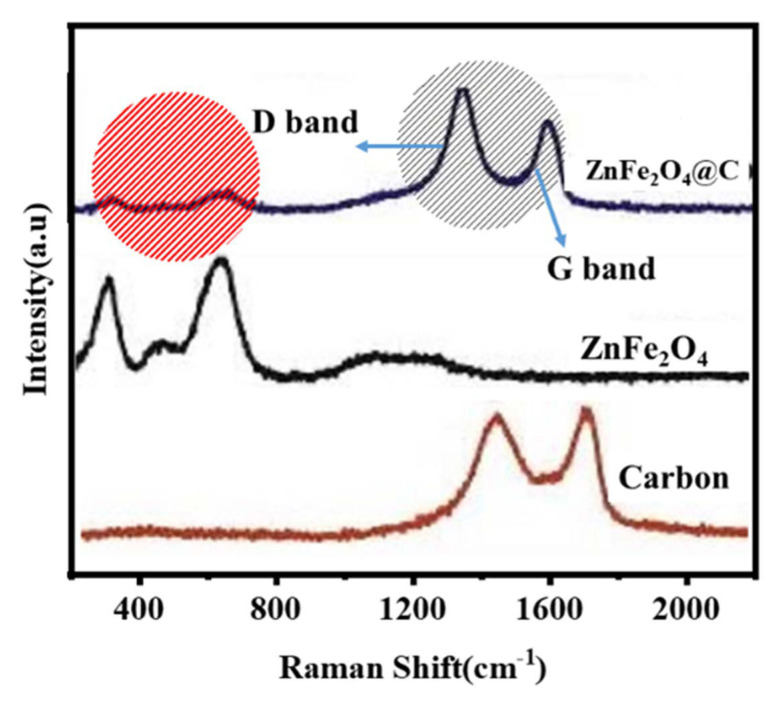
The Raman spectra of carbon, ZnFe_2_O_4_ and ZnFe_2_O_4_@C.

Raman spectroscopy is a quick and easy method for finding more information about carbon. Carbon, ZnFe_2_O_4_ nanoparticles and ZnFe_2_O_4_@C nanohybrids are shown in Figure 3. Carbon and ZnFe_2_O_4_@C nanohybrids have G and D bands. The G band at about 1580 cm^−1^, which corresponds to an E2g mode of graphite, comes from the vibration of sp2-bonded carbon atoms. The D band at about 1345 cm^−1^, which corresponds to a point mode of A1g symmetry, comes from the defects and disorder of carbon materials. The ID/IG of ZnFe_2_O_4_@C is higher than that of carbon, which is due to the smaller average size of sp2 domains and more disordered degrees and defects. This is because sp2 domains are smaller, and there are more defects. The increase in ID/IG also shows that GO is turned into RGO during the reaction process. There are a lot of Raman bonds in ZnFe_2_O_4_ and ZnFe_2_O_4_@C that look a lot like the bonds in ZnFe_2_O_4_ particles and ZnFe_2_O_4_@C nanocomposites that have been reported before. The Raman spectra of ZnFe_2_O_4_ and graphene show that the properties of ZnFe_2_O_4_@C stay the same even after they are made into nanohybrids [36,37].

**Figure 4:**
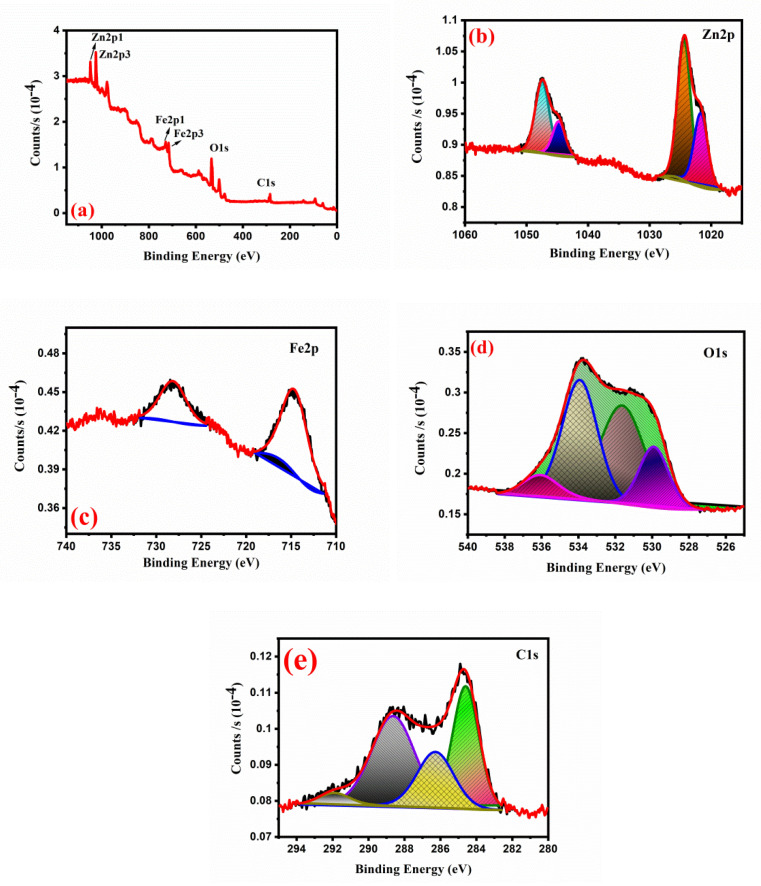
The XPS spectra of ZnFe_2_O_4_: (**a**) survey spectrum; (**b**) Zn 2p spectrum (**c**); Fe 2p spectrum; (**d**) O1s spectrum; (**e**) C 1s spectrum.

XPS spectra were used to understand the chemical composition. Figure 4a shows an in situ carbon ZnFe_2_O_4_-coated survey spectrum that represents Zn, Fe, O and C characteristics, respectively. As represented in Figure 4b, the spectrum of Zn 2p is shown. The peaks at 1022.5 and 1045.4 eV correspond to Zn 2p_3/2_ and Zn 3p_1/2_, respectively. The XPS spectrum of Fe 2p was shown in Figure 4c. The orbital pairs Fe 2p_1/2_ and Fe 2p_3/2_ are characterized as peaks of 724 and 711 eV. Figure 4d shows the XPS spectrum of O 1s. The top at 530 eV, from left to right, indicates that the functional oxygen group and the peak at 536 are associated with large oxygen molecules on the surface of samples. Figure 4e shows that the peak of C1s spectra was observed. The maximum of 285 eV is C=C; the maximum of 285 eV is C=H and the maximum fitness is C=N and C–OH. The successful preparation of in situ carbon-coated ZnFe_2_O_4_ has been proven once more.

Nuli et al. found that Li^+^ was reversibly embedded and eliminated in ZnFe_2_O_4_ [36]. In 2010, Guo et al. discussed the Li storage mechanism of ZnFe_2_O_4_ as follows [35,37].

ZnFe_2_O_4_ + xLi^+^ + xe^−^ → Li_x_ZnFe_2_O_4_ (1)

Li_x_ZnFe_2_O_4_ + (8 − x)Li^+^ + (8 − x)e^−^ → Zn^0^ + 4Li_2_O + 2Fe^0^ (2)

Zn^0^ + Li^+^ + e^−^
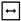
LiZn (3) Zn^0^ + Li_2_O → ZnO + 2Li + 2e^−^(3)

2Fe^0^ + 3Li_2_O → Fe_2_O_3_ + 2Li ^+^ + 2e^−^ (4)

**Figure 5:**
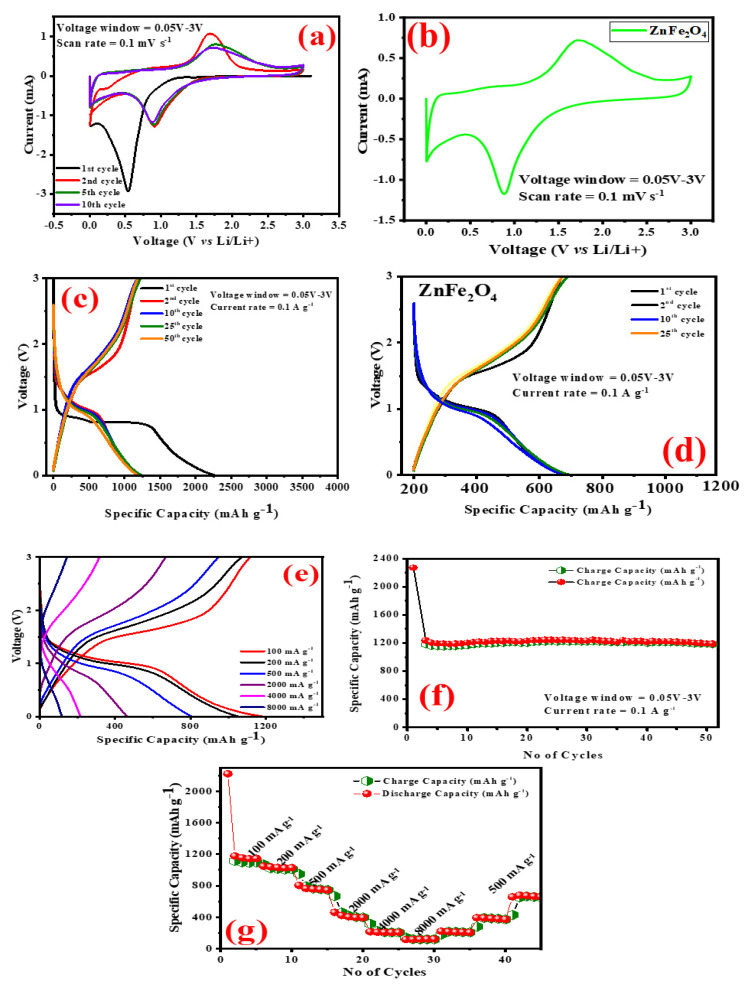
The electrochemical performance. (**a**) Cyclic Voltametry of carbon coated ZnFe_2_O_4_. (**b**) Cyclic Voltametry of pure ZnFe_2_O_4_; (**c**) charge/discharge profile of carbon coated ZnFe_2_O_4_; (**d**) charge/discharge profile of pure ZnFe_2_O_4_; (**e**) charge/discharge profile of carbon coated ZnFe_2_O_4_ at different current densities; (**f**) the cyclic performance; (**g**) rate performance of the electrode.

In order investigate the electrochemical mechanism during charging and discharging, CV analysis was performed to characterize the sample’s electrochemical characteristics. The CV curves of carbon-coated ZnFe_2_O_4_ for the first 10 cycles was shown in Figure 5a. The voltage range is 0.05–3 V at a rate of 0.1 mV s^−1^. As seen in Figure 5a, in the first cycle of cathodic scanning, carbon-coated ZnFe_2_O_4_ shows a sharp reduction peak at 0.5–0.6 V. This was largely attributable to decreases in Fe^3+^ and Zn^2+^ to Fe and Zn and Li-Zn and Li_2_O, the mechanism of Li-ion intercalation in accordance with Equations (1)–(4) [38]. After subsequent cycles of scanning, the reduction peaks of carbon-coated ZnFe_2_O_4_ shifted to 1.0 V, which is due to changes in the internal structure of carbon coated ZnFe_2_O_4_. When the first cycle was observed, a wide reduction peak of 1.6 V was observed due to the oxidation of Zinc to Zn^2+^ and iron oxide to Fe^3+^, the mechanism for Li intercalation/de-intercalation [39]. Previous results of ZnFe_2_O_4_ [40] show that the peak area and current of carbon-coated ZnFe_2_O_4_ possess enhanced kinetics and faster ion and faster transport of electrons, which results in better electrochemical performance. The CV curve of pure ZnFe_2_O_4_ is shown in Figure 5b.

For further investigation, the prepared samples are assembled into a coin cell and tested at a 0.05–3 V range of constant current load/discharge (0.1 Ag^−1^). Figure 5c shows the charge/discharge curves of carbon-coated ZnFe_2_O_4_ for 50 cycles. During the process of charging/discharging, the voltage plateau was observed, which corresponds to a redox reaction of the sample. On the first discharge curve observation of carbon-coated ZnFe_2_O_4_, an obvious working plateau at 0.8 V was observed. After a few cycles, the plateau disappears and starts slopping, which matches with the results of CV in Figure 5a. Due to the oxidation of Zn to Zn^2+^ and Fe to Fe^3+^, a plateau was observed at 1.6 V. As per the charge/discharge curves (Figure 5b), the 25th and 50th cycles coincide with another, which shows the enhanced stability of the carbon-coated ZnFe_2_O_4_ electrode. The first discharge capacity of the sample reaches 2267 mAh·g^−1^ at 0.1 Ag^−1^ and The capacity for the first load is 1221 mAh·g^−1^. The charge/discharge curves of different current densities (100–8000 mA·g^−1^) are shown in Figure 5c. The initial capacity loss may be due to lithiation, which consumes irreversible Li ion and results in the formation of solid electrolyte interphases [41]. The sample attained an efficiency of about 96% after a few cycles, indicating carbon-coated ZnFe_2_O_4_ with good electrochemical performances. The charge/discharge profile of pure ZnFe_2_O_4_ is shown in Figure 5d.

Figure 5e represents the cyclic curves of carbon-coated ZnFe_2_O_4_ tested at a current density of 100 mA·g^−1^. The sample is tested in the same voltage window (0.05–3 V) for all electrochemical analyses. Wang et al. [42] reported that pure ZnFe_2_O_4_ has a 1312 mAh·g^−1^ reversible capacity and 100 cycles have reduced the discharge capacity toward 361 mAh·g^−1^. The reason for capacity loss is due to the poor electronic conductivity of ZnFe_2_O_4_. Comparing carbon-coated ZnFe_2_O_4_ with pure ones, the electrochemical performance of the carbon-coated sample was greatly improved. The discharge capacity of ZnFe_2_O_4_ carbon coated reaches 1312 mAh·g^−1^ in the first cycle and maintained 1228 mAh·g^−1^ after 50 cycles. The high capacity is due to (i) carbon as a conducting layer in a composite that enhances electron transport and (ii) in situ carbon-coated defects on ZnFe_2_O_4_ surface are present in chemical oxidation, where more Li ions are stored. Thus, the contact area between electrode/electrolyte increases, and during intercalation/de-intercalation, Li ion/electron movements accelerate. (iii) Carbon has a mechanical resilience in the exterior layer, reducing the expansion in volume during electrical processes. The reversible capacities of carbon-coated ZnFe_2_O_4_ increases with the number of cycles. This is a common critical feature of LIBs anode transition metal oxides. The reason is due to the gradual activation of metal oxides and electrolytes and the reversible mechanism [35,43].

In order to further observe the cycling performance of carbon-coated ZnFe_2_O_4_ at different current densities, electrode tests were conducted for rate performance. Current density increased from 100 to 8000 mAh·g^−1^ and then back to 500 mA·g^−1^ respectively. As shown in Figure 5f,g, at the current densities of 100, 200, 500, 2000, 4000 and 8000 mA·g^−1^, the discharge capacity is about 1312, 1059, 806, 471, 207 and 119 mAh·g^−1^. It then return to 500 mA·g^−1^, and the discharge capacity still reaches 645 mAh·g^−1^, which shows the good reversibility nature of carbon-coated ZnFe_2_O_4_. Compared with pure ZnFe_2_O_4_ [44] without carbon coating, the performance of carbon-coated ZnFe_2_O_4_ is greatly improved. The previous reported studies [45,46,47,48,49,50,51,52,53,54,55,56,57,58,59,60,61,62,63] are shown in Table 1.

**Figure 6:**
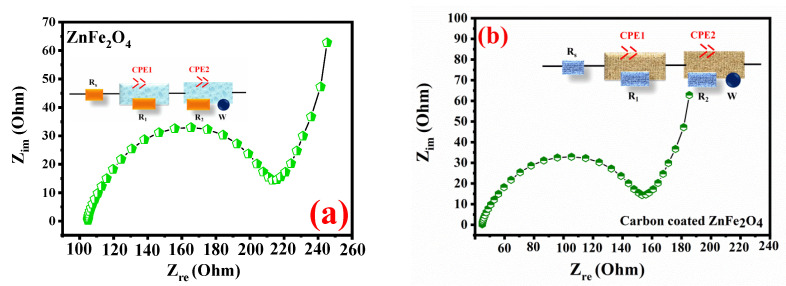
The impedance spectra of (**a**)ZnFe_2_O_4_. (**b**) Carbon-coated ZnFe_2_O_4_.

Figure 6 shows the EIS spectra of carbon-coated ZnFe_2_O_4_ and displays anode materials’ load transmission resistance. The spectrum consists of half of the circle and an inclining line, as illustrated in Figure 6a,b. The half circle in the high frequency region features resistance toward the charge transfer of the electrode to the electrolyte. The low frequency slope shows an impedance in Warburg, which is the diffusion of lithium ion in electrodes [64,65,66,67]. Compared with pure ZnFe_2_O_4_ (Figure 6a), the resistance of carbon-coated ZnFe_2_O_4_ is smaller. Carbon-coated ZnFe_2_O_4_ shows faster ion transfer because the addition of carbon increases the electronic conductivity of carbon-coated ZnFe_2_O_4_, thus improveing electrochemical performances. Electrochemical and the impedance results show that carbon-coated ZnFe_2_O_4_ have enhanced electrochemical performance compared to anode materials.

## 3. Conclusions

In the present study, an electrochemical investigation was conducted on carbon-coated ZnFe_2_O_4_ as an anode for energy storage applications. The in situ carbon-coated ZnFe_2_O_4_ with spherical structure was prepared by using the sol–gel technique. Based on previous reports on ZnFe_2_O_4_’s poor stability, electronic conductivity and electrochemical performances have been improved on the surface of ZnFe_2_O_4_’s sphere structure with carbon coating. As a result, it is observed that the discharge capacity of ZnFe_2_O_4_ is 1312 mAh·g^−1^ at 100 mA·g^−1^, and the capacity retention is 95% after 50 cycles. The above results show that cycling and the rate performance of ZnFe_2_O_4_ carbon-coated was enhanced by the addition of carbon. The electrochemical performance of carbon-coated ZnFe_2_O_4_ is suitable for enhanced anode materials for Li-ion batteries.

## 4. Experimental

**Figure 7:**
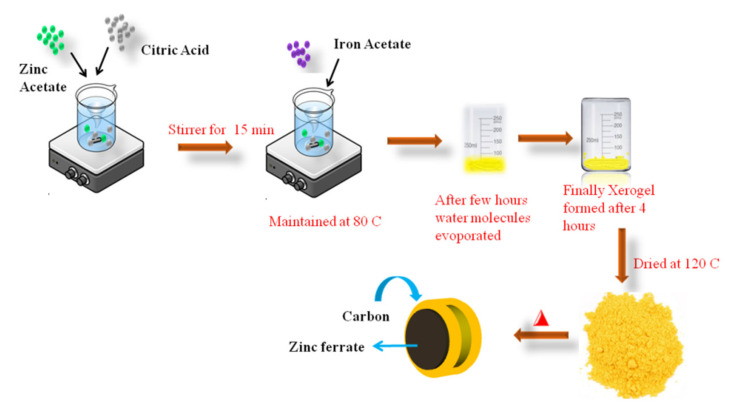
The preparation of in situ carbon-coated ZnFe_2_O_4_ by the sol–gel method.

The nanocrystalline powders of ZnFe_2_O_4_ are synthesized by means of the conventional sol–gel assisted combustion method. This is carried out in two stages in which the xerogel is initially prepared using the sol–gel method, followed by the combustion method at high temperatures. The calculated amounts of zinc acetate, iron acetate and citric acid are mixed together in 100 mL of distilled water. After that, the pH of the solution is carefully controlled at 7 by using ammonia water. Here, citric acid is used as both a chelating agent and carbon source. The resulting solution is constantly maintained at 80 °C in a stirrer until the water molecules evaporated. The resulting xerogel transforms into a fluffy powder while drying at 120 °C for 12 h. Finally, the sample is calcinated at 600 °C for 4 h to obtain the final sample. The preparation procedures are shown in Figure 7

### 4.1. The Electrochemical Studies

The battery tests are performed using CR2032 coin cells in an argon atmosphere inside a glove box. The cathode was the prepared sample, the anode comprised Li metal and the separator was polypropylene. The electrolyte was constructed by combining LiPF6 with EC and DEC (1:1 *v*/*v*). The cathodes were made by combining 2.5 g of prepared active material with 0.5 g of ketjen black and 0.5 mg of teflonized acetylene black (TAB-2). Before fabricating coin cells, the prepared mixture was pasted on a stainless-steel current collector and dried in a vacuum oven at 160 °C. The charge–discharge cycle was carried out using the Arbin BT–2000 battery tester system. An electrochemical workstation was used to conduct EIS analyses (SP-150, Biologic, Seyssinet-Pariset, France).

### 4.2. Characterization Details

Full-Prof software is used to calculate crystal values and structural analysis by X-ray diffraction (Cu K radiation, Rigaku, Tokyo, Japan). A scanning electron microscope (Hitachi, Tokyo, Japan) coupled with an EDX module and a high-resolution transmission electron microscope were used to examine surface morphology and elemental composition (HRTEM, JEOL, Tokyo, Japan). The molecular structures of the material were analyzed by using Nuclear Magnetic Resonance Spectrometer (NMR; HWB NMR, Birmingham, UK).

## Figures and Tables

**Table 1:** Comparison of cycling performance with different ZnFe_2_O_4_-based electrodes by synthesis methods.

Electrode Materials	Synthesis Method	Current mA·g^−1^	Cycle	Discharge Capacity mAh·g^−1^
3D Porous ZnFe_2_O_4_	Sol–Gel	1000	400	711 [45]
ZnFe_2_O_4_ Nanofibers	Electro Spinning	50	50	1142 [46]
N-doped Carbon coated ZnFe_2_O_4_	Electro Spinning	200	200	881 [47]
ZnFe_2_O_4_ C/N Doped graphene	Hydrothermal Method	100	100	952 [48]
ZnFe_2_O_4_/double graphene	Microwave irradiation	1000	200	475 [49]
Porous ZnFe_2_O_4_	Hydrothermal Method	200	80	868 [50]
ZnFe_2_O_4_/C	Ionic Liquid	500	190	1091 [51]
Acetylene Black/ZnFe_2_O_4_/C	Thermal Decomposition	1000	200	430 [52]
ZnFe_2_O_4_/hollow fiber	Electro spinning	200	260	1026 [53]
ZnFe_2_O_4_ Nanorods	Co-Precipitation	100	50	983 [28]
ZnFe_2_O_4_@C/graphene	Hydrothermal Method	250	180	705 [54]
3D- ZnFe_2_O_4_/Graphene	Hydrothermal Method	100	50	770 [55]
ZnFe_2_O_4_ Nanosphere/G	Solvothermal	100	50	704 [31]
ZnFe_2_O_4_/Graphene	Cathodic Deposition	200	200	881 [56]
ZnFe_2_O_4_/Nanoflake/g	Hydrothermal Method	100	100	730 [57]
Carbon Coated ZnFe_2_O_4_ Nanowires	Micro-Emulsion	100	100	1292 [58]
ZnFe_2_O_4_/C	Planetary Ball-Mill	100	60	1100 [59]
ZnFe_2_O_4_/Graphene	Hydrothermal Method	100	50	956 [60]
ZnFe_2_O_4_/C	Planetary Ball-Mill	400	160	1300 [61]
MWCNT/ZnFe_2_O_4_	High-Temperature	60	50	1152 [62]
ZnFe_2_O_4_ Nano-Octahedral	Hydrothermal Method	1000	300	730 [25]
ZnFe_2_O_4_/Graphene	Solvothermal	400	90	398 [63]
ZnFe_2_O_4_ Nanofibers	Electro spinning	60	30	733 [32]
In situ ZnFe_2_O_4_/C	Sol–Gel	100	50	1312 (This Work)

## Data Availability

Not applicable.
